# Time of the low-level cardiopulmonary exercise test does not affect the evaluation of acute myocardial infarction in stable status

**DOI:** 10.3389/fcvm.2022.1092787

**Published:** 2022-12-20

**Authors:** Lanting Zhao, Yuanwei Liu, Siyuan Li, Ying Xie, Yajun Xue, Yifang Yuan, Rong He, Fei She, Tingting Lv, Ping Zhang

**Affiliations:** ^1^Department of Cardiology, Beijing Tsinghua Changgung Hospital, Beijing, China; ^2^Peking University Clinical Research Center, Peking University First Hospital, Beijing, China; ^3^Department of Epidemiology and Biostatistics, Peking University School of Public Health, Beijing, China

**Keywords:** acute myocardial infarction, cardiopulmonary exercise test (CPET), time, cardiac rehabilitation, efficiency

## Abstract

**Introduction:**

Cardiopulmonary exercise test (CPET) provides the means to evaluate the cardiopulmonary function and guide cardiac rehabilitation. The performance of acute myocardial infarction (AMI) patients at different times is different on CPET.

**Materials and methods:**

This was a cross-sectional study. Patients diagnosed as AMI in stable status were included and performed the low- level CPET (RAMP 10W). CPET variables at different times were compared among four groups.

**Results:**

Sixty and one patients with AMI conducted the low-level CPET from 3 to 15 days after AMI. Patients were stratified according to quartiles of CPET's time: 5 in 3–6 days group, 34 in 7–9 days group, 14 in 10–12 days group, 8 in 13–15 days group. Only VO2/HR at rest showed statistically different among the four groups.VO2/HR at rest in 3–6 days group and 10–12 days group were higher than in 13–15 days group (3.4 ± 0.85, 3.18 ± 0.78 vs. 2.50 ± 0.49 ml/beat, *p* < 0.05). Patients with complete revascularization had higher peak heart rate and blood pressure product and peak breathing reserve (BR), and lower Borg score compared with incomplete revascularization. And patients with LVEF >50% had higher peak BR compared with LVEF 40–50%.

**Conclusion:**

It was safe and efficient to conduct the low-level CPET in stable AMI patients 3 days after onset. Time was not an effector on cardiopulmonary function and exercise capacity and prognosis in AMI during CPET. Complete revascularization and normal LVEF should be good for exercise test in AMI.

## Introduction

Acute myocardial infarction (AMI) has become an important cause of emergency medical treatment, readmission, and death (1). Cardiac rehabilitation (CR), including exercise training, lifestyle intervention, and health education is recommended as the Class I for patients with AMI (2–5). Exercise rehabilitation improves physical activity levels (6) and modifiable cardiovascular risk factors, including abdominal adiposity, impaired glucose tolerance, hypertension, low serum high-density lipoprotein cholesterol (LDL-C), and hypertriglyceridemia in AMI patients (7–9).

Cardiopulmonary exercise test (CPET), a non-invasive and safe approach to evaluate exercise capacity and cardiopulmonary function, plays an irreplaceable role in rehabilitation guidance, treatment effect assessment, and prognosis evaluation (10–14). CPET has been widely used in early AMI to evaluate prognosis and guide exercise training (15–17). It is reported that the time of CPET in AMI ranges from 3 days to 7 weeks after AMI, including in hospital and after discharge (18–22). The performance of AMI patients on different time's CPET is different, however, the effect of CPET's time is not well recognized. The purpose of this study is to explore the effect of CPET's time on the performance of AMI patients and the efficacy and safety of CPET at different times.

## Materials and methods

### Study population

This was a cross-sectional study that included patients diagnosed as AMI and performed CPET in Beijing Tsinghua Changgung Hospital from June 2018 to March 2019. Patients with AMI were firstly treated with the emergency coronary intervention, then performed CPET when in stable status. The stable AMI patients were those (1) no chest pain; (2) no further increase in myocardial injury markers; (3) no manifestation of decompensated heart failure (HF); (4) no new ischemic symptoms and signs; (5) no severe arrhythmia. AMI patients with severe comorbidities (e.g., infection, thromboembolism, respiratory failure, etc.) or complications (e.g., severe arrhythmia, cardiogenic shock, HF [left ventricular ejection fraction (LVEF) <40%, etc.,] performed CPET after the condition were stable. All patients were provided and signed CPET informed consent. The study was approved by the Beijing Tsinghua Changgung Hospital Research Ethics Committee (18084-0-01) that followed for the World Medical Association Declaration of Helsinki.

### Cardiopulmonary exercise test

CPET was performed on a cycle ergometer (Miraclink-200P, China) in an air-conditioned room. Patients with AMI underwent the low-level CPET, namely RAMP10W scheme. Patients firstly sit for 3 min, then cycled without load for 3 min and continued with the incremental work rate of 10 W/min until the peak respiratory exchange rate (RER) reached 1.0, or Borg score got 12–13 points, or the patient requested to stop. During the exercise period, the speed of the treadmill was required to maintain 60 rpm. Patients finally had a rest for 8 min. The 12-lead electrocardiogram, heart rate (HR), and blood pressure were continuously monitored using an automated sphygmomanometer (Tango M2, SunTech, USA) every 2 min during the test. Inhaled and expired gasses were collected by a face mask every 3 s and analyzed breath-by-breath using the Geratherm Respiratory (Ergostik, Blue Cherry Software, Germany). Because the purpose of the test was to guide the CR in AMI, CPET was generally stopped around the anaerobic threshold (AT) by the cardiologists. The peak oxygen uptake (VO2) was the average value of the highest value during the last 30 s of exercise, and AT was determined by the slope method or the ventilation equivalent method (14). Gas flow and concentration calibration were corrected daily before the test.

### Statistical analysis

All data were analyzed using SPSS 21.0 software. Measurement data were expressed as mean ± standard deviation. Comparison between groups was tested by one-way analysis of variance and *t-*test, and comparison of count data was analyzed by the chi-square test. *P* < 0.05 was considered statistically significant.

## Results

### General clinical characteristics

**Figure 1:**
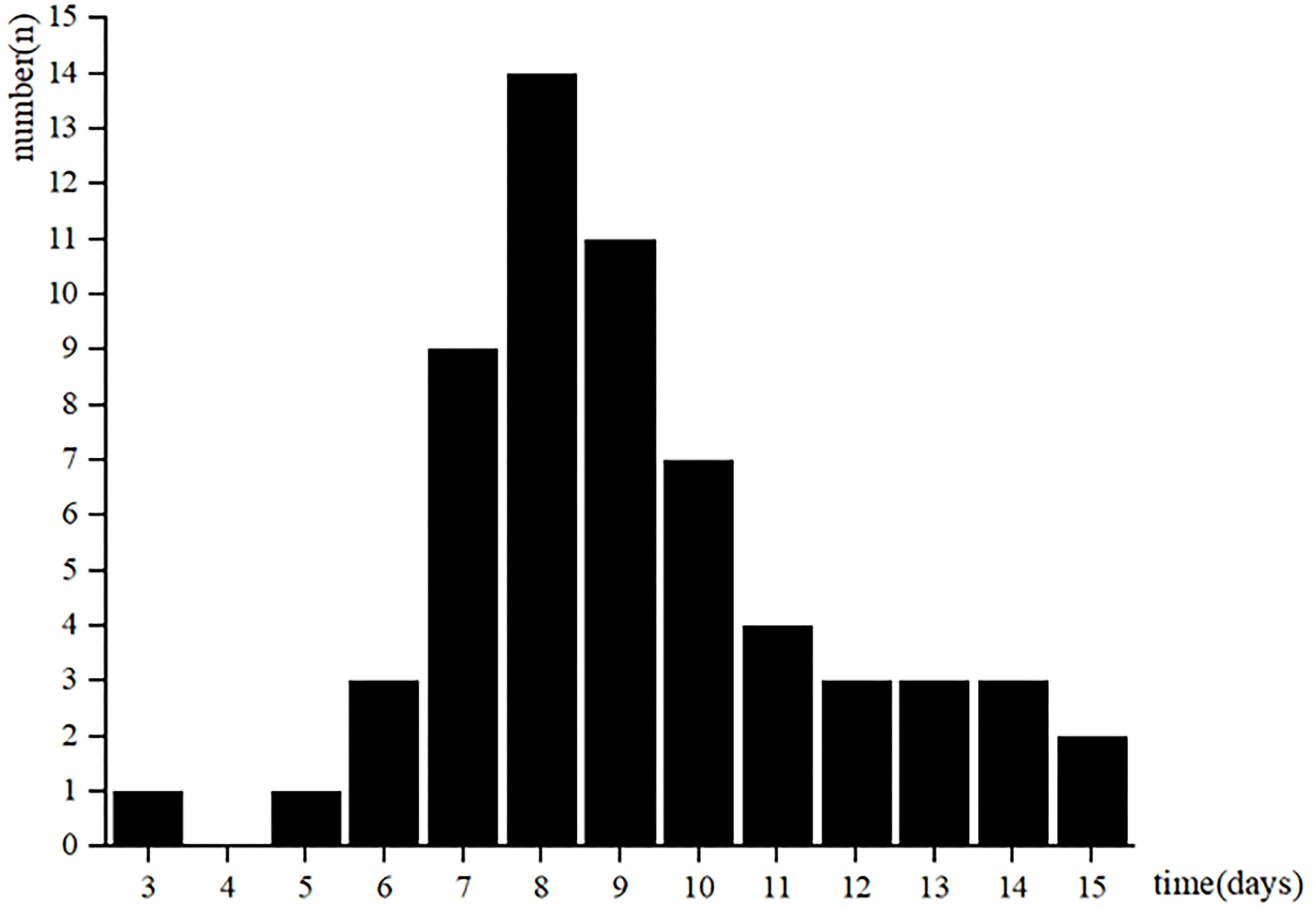
The distribution of CPET time.

A total of 61 patients with AMI in stable status performed CPET. The low-level CPET was conducted from 3 to 15 days after AMI (Figure 1). Patients were stratified according to quartiles of the CPET's times: 5 patients in 3–6 days group, 34 patients in 7–9 days group, 14 patients in 10–12 days group, 8 patients in 13–15 days group. There was no statistical difference among the four groups in baseline clinical characteristics (Table 1).

**Table 1:** General clinical characteristics.

	**3–6 days**	**7–9 days**	**10–12 days**	**13–15 days**	***P*-value**
	**(*n* = 5)**	**(*n* = 34)**	**(*n* = 14)**	**(*n* = 8)**	
Days	5.20 ± 1.30	8.10 ± 0.80	10.70 ± 0.80	13.88 ± 0.80	0.00
Age (years)	52.00 ± 19.10	59.53 ± 9.66	60.64 ± 10.68	61.50 ± 10.70	0.42
Man (%)	4 (80.0%)	28 (82.4%)	11 (78.6%)	5 (62.5%)	0.68
BMI (kg/cm^2^)	27.40 ± 4.56	25.88 ± 3.57	24.86 ± 3.06	25.75 ± 3.15	0.56
**Risk factors** ***n*** **(%)**					
Smoking history	3 (60.0%)	24 (70.6%)	10 (71.4%)	4 (50.0%)	0.69
Hypertension	3 (60.0%)	19 (55.9%)	10 (71.4%)	4 (50.0%)	0.75
Diabetes mellitus	1 (20.0%)	8 (23.5%)	4 (28.6%)	3 (37.5%)	0.85
Dyslipidemia	2 (40.0%)	26 (76.5%)	10 (71.4%)	7 (87.5%)	0.32
**Medications** ***n*** **(%)**					
ACEI/ARB	4 (80.0%)	27 (79.4%)	12 (85.7%)	8 (100.0%)	0.64
Beta blocker	5 (100.0%)	28 (82.4%)	13 (92.9%)	8 (100.0%)	0.59
Elective revascularization *n* (%)	2 (40.0%)	9 (26.5%)	3 (21.4%)	1 (12.5%)	0.79
LVEF (%)	58.80 ± 8.64	56.44 ± 8.61	56.14 ± 7.12	58.38 ± 7.31	0.86

BMI, body mass index; ACEI, angiotensin converting enzyme inhibitor; ARB, angiotensin receptor blocker; LVEF, center ventricular ejection fraction.

### CPET variables changes at different times

CPET variables were compared among the four groups, and only VO2/HR at rest showed statistically different (Table 2). VO2/HR at rest in 3–6 days group and 10–12 days group were higher than in 13–15 days group (3.4 ± 0.85, 3.18 ± 0.78 vs. 2.50 ± 0.49 ml/beat, *p* < 0.05). However, other CPET variables, like VO2 at AT, VO2/HR at AT, VO2/WR slope, VE/VCO2 slope, oxygen uptake efficiency slope (OUES), etc., showed no statistical difference among the four groups.

**Table 2:** CPET variables in different times.

**Variables**	**3–6 days (*n* = 5)**	**7–9 days (*n* = 34)**	**10–12 days (*n* = 14)**	**13–15 days (*n* = 8)**	**Average**	***P*-value**
WR peak (watt)	77.60 ± 37.16	70.35 ± 18.41	67.71 ± 16.68	65.50 ± 26.38	69.70 ± 20.70	0.76
HR rest (bpm)	71.00 ± 4.64	73.50 ± 9.70	74.21 ± 7.44	73.63 ± 6.84	73.48 ± 8.45	0.91
HR AT (bpm)	97.60 ± 6.95	97.15 ± 12.93	96.86 ± 7.39	99.38 ± 16.35	97.41 ± 11.78	0.97
HR peak (bpm)	106.20 ± 10.57	107.00 ± 14.62	105.86 ± 9.54	108.12 ± 19.27	106.82 ± 13.73	0.99
SBP rest (mmHg)	125.00 ± 9.97	156.00 ± 189.60	126.71 ± 16.54	125.88 ± 14.54	142.79 ± 141.72	0.89
DBP rest (mmHg)	75.80 ± 10.45	73.21 ± 10.98	70.79 ± 14.05	72.00 ± 8.30	72.70 ± 11.23	0.84
SBP peak (mmHg)	147.20 ± 34.87	165.71 ± 31.56	157.29 ± 21.92	162.62 ± 16.43	161.85 ± 28.21	0.51
DBP peak (mmHg)	83.40 ± 16.68	75.97 ± 12.60	73.43 ± 11.84	68.62 ± 12.32	75.03 ± 12.90	0.22
VO2/HR rest (ml/beat)	3.40 ± 0.85	2.89 ± 0.52	3.18 ± 0.78	2.50 ± 0.49	2.95 ± 0.64	0.03
VO2/HR AT (ml/beat)	9.42 ± 1.47	7.73 ± 1.83	8.17 ± 2.32	7.40 ± 2.08	7.94 ± 1.98	0.28
VO2/HR peak (ml/beat)	10.34 ± 1.90	8.61 ± 2.22	9.04 ± 2.35	7.97 ± 2.48	8.77 ± 2.28	0.30
CO rest (L/min)	3.04 ± 0.58	2.58 ± 0.51	2.84 ± 0.70	2.26 ± 0.47	2.63 ± 0.59	0.05
CO AT (L/min)	6.76 ± 2.16	5.28 ± 1.39	5.52 ± 1.42	5.00 ± 1.39	5.43 ± 1.49	0.18
CO peak (L/min)	7.40 ± 2.43	5.69 ± 1.65	5.92 ± 1.51	5.10 ± 1.77	5.80 ± 1.74	0.12
CO peak pre (%)	48.80 ± 15.16	38.24 ± 9.28	40.50 ± 8.01	36.12 ± 7.70	39.34 ± 9.66	0.09
VO2 AT (ml/kg/min)	11.54 ± 2.15	9.75 ± 1.93	10.14 ± 2.43	9.09 ± 0.80	9.90 ± 2.01	0.17
VO2 peak (ml/kg/min)	14.52 ± 3.83	12.26 ± 2.55	12.17 ± 2.65	10.30 ± 1.66	12.17 ± 2.71	0.05
VO2 peak pre (%)	52.40 ± 10.71	49.44 ± 13.45	48.64 ± 12.11	43.75 ± 11.68	48.75 ± 12.63	0.63
VE/VCO2 slope	29.68 ± 3.61	30.99 ± 8.59	30.64 ± 3.70	28.09 ± 4.46	30.42 ± 6.91	0.76
VO2/WR slope	10.20 ± 1.22	8.34 ± 1.81	8.56 ± 1.33	8.21 ± 1.36	8.52 ± 1.66	0.12
OUES	1.71 ± 0.77	1.61 ± 0.44	1.65 ± 0.53	1.61 ± 0.45	1.63 ± 0.48	0.97
HR/VO2 slope	2.94 ± 1.13	3.46 ± 1.18	3.95 ± 1.56	4.19 ± 2.81	3.62 ± 1.55	0.39
DP peak (bpm*mmHg)	15,698.00 ± 4,554.16	17,803.35 ± 4,452.77	16,768.71 ± 3,474.02	17,736.12 ± 4,153.59	17,384.51 ± 4,166.14	0.69
Borg	13.20 ± 1.10	14.21 ± 1.90	13.71 ± 1.68	12.88 ± 1.89	13.84 ± 1.83	0.24
RER rest	0.88 ± 0.04	0.86 ± 0.06	0.82 ± 0.18	0.88 ± 0.06	0.85 ± 0.10	0.58
RER AT	0.92 ± 0.08	0.90 ± 0.06	0.93 ± 0.08	0.94 ± 0.06	0.92 ± 0.07	0.42
RER peak	1.04 ± 0.19	0.98 ± 0.07	0.98 ± 0.09	1.02 ± 0.10	0.99 ± 0.09	0.44
BR peak (%)	60.00 ± 11.31	59.21 ± 15.08	64.43 ± 8.32	58.75 ± 20.58	60.41 ± 14.24	0.70
VE AT (L/min)	28.40 ± 4.51	25.91 ± 5.00	26.07 ± 7.70	21.88 ± 5.11	25.62 ± 5.81	0.20
VE peak (L/min)	37.40 ± 7.96	33.53 ± 9.47	32.50 ± 9.80	27.38 ± 6.63	32.80 ± 9.25	0.24

WR, work rate; HR, heart rate; AT, anaerobic threshold; SBP, systolic blood pressure; DBP, diastolic blood pressure; VO2, oxygen uptake; CO, cardiac output; VE, ventilation; VCO2, carbon dioxide output; OUES, oxygen uptake efficiency slope; DP, heart rate and blood pressure product; RER, respiratory exchange rate.

### CPET variables changes at the different cardiac status

Further, to analysis the effect of cardiac factors on CPET variables, all patients were divided to complete revascularization group and incomplete revascularization group; LVEF 40–50% group and LVEF >50% group (Table 3). Moreover, complete revascularization referred to that all stenotic vessels >70% stenosis were revascularized when patients performed the CPET. Peak HR and blood pressure product (DP) in complete revascularization group was higher than in the incomplete revascularization group (18,121.89 ± 4,236.16 vs. 15,123.20 ± 3,072.48 bpm ^*^ mmHg, *p* < 0.05). Borg score in incomplete revascularization group was higher than in complete revascularization group (14.93 ± 1.44 vs. 13.48 ± 1.81, *p* < 0.05) and breathing reserve (BR) at peak in incomplete revascularization group was lower than in complete revascularization group (53.00 ± 16.19 vs. 62.83 ± 12.83%, *p* < 0.05). And BR at peak in LVEF 40–50% group was lower than in LVEF >50% group (54.72 ± 15.62 vs. 62.79 ± 13.09%, *p* < 0.05). Other CPET variables showed no statistical difference between the two cardiac factors groups.

**Table 3:** CPET variables in different groups.

**Variables**	**Complete revascularization (*n* = 46)**	**Not complete revascularization (*n* = 15 VO)**	***p*-value**	**LVEF 40–50% (*n* = 28)**	**LVEF >50% (*n* = 33)**	***P*-value**
WR peak (watt)	73.73 ± 19.67	68.39 ± 21.07	0.39	72.33 ± 21.03	68.60 ± 20.71	0.53
HR rest (bpm)	73.33 ± 8.54	73.52 ± 8.51	0.94	74.22 ± 8.57	73.16 ± 8.48	0.66
HR AT (bpm)	93.13 ± 8.29	98.80 ± 12.47	0.11	98.06 ± 11.07	97.14 ± 12.18	0.78
HR peak (bpm)	100.80 ± 11.71	108.78 ± 13.89	0.05	108.61 ± 13.24	106.07 ± 14.02	0.51
SBP rest (mmHg)	123.80 ± 9.21	148.98 ± 163.08	0.55	120.33 ± 16.78	152.19 ± 168.14	0.43
DBP rest (mmHg)	70.73 ± 12.06	73.35 ± 11.02	0.44	70.17 ± 11.96	73.77 ± 10.88	0.26
SBP peak (mmHg)	149.87 ± 22.82	165.76 ± 28.91	0.06	151.89 ± 28.69	166.02 ± 27.27	0.07
DBP peak (mmHg)	75.13 ± 10.51	75.00 ± 13.69	0.97	74.11 ± 10.65	75.42 ± 13.83	0.72
VO2/HR rest (ml/beat)	3.06 ± 0.77	2.91 ± 0.60	0.44	2.88 ± 0.54	2.98 ± 0.69	0.59
VO2/HR AT (ml/beat)	8.50 ± 2.34	7.75 ± 1.83	0.21	7.95 ± 1.75	7.94 ± 2.08	0.98
VO2/HR peak (ml/beat)	9.51 ± 2.45	8.52 ± 2.19	0.14	8.96 ± 2.09	8.69 ± 2.37	0.68
CO rest (L/min)	2.81 ± 0.67	2.58 ± 0.55	0.18	2.74 ± 0.68	2.59 ± 0.55	0.37
CO AT (L/min)	5.81 ± 1.75	5.30 ± 1.39	0.26	5.74 ± 1.73	5.31 ± 1.39	0.33
CO peak (L/min)	6.27 ± 1.87	5.65 ± 1.69	0.23	6.10 ± 1.99	5.68 ± 1.64	0.39
CO peak pre (%)	38.07 ± 9.00	39.76 ± 9.92	0.56	38.78 ± 9.35	39.58 ± 9.89	0.77
VO2 AT (ml/kg/min)	10.06 ± 2.34	9.85 ± 1.92	0.72	9.89 ± 1.81	9.90 ± 2.12	0.98
VO2 peak (ml/kg/min)	12.14 ± 3.06	12.18 ± 2.62	0.96	11.98 ± 2.40	12.25 ± 2.85	0.72
VO2 peak pre (%)	48.47 ± 10.41	48.85 ± 13.38	0.92	50.22 ± 12.40	48.14 ± 12.82	0.56
VE/VCO2 slope	33.18 ± 11.58	29.52 ± 4.31	0.07	30.66 ± 4.33	30.32 ± 7.78	0.86
VO2/WR slope	8.07 ± 2.01	8.67 ± 1.53	0.23	8.37 ± 1.81	8.59 ± 1.62	0.64
OUES	1.54 ± 0.60	1.66 ± 0.44	0.42	1.61 ± 0.49	1.64 ± 0.49	0.86
HR/VO2 slope	3.03 ± 1.60	3.82 ± 1.50	0.09	3.89 ± 1.41	3.51 ± 1.61	0.39
DP peak (bpm*mmHg)	15,123.20 ± 3,072.48	18,121.89 ± 4,236.16	0.01	16,516.67 ± 3,872.47	17,747.79 ± 4,274.06	0.30
Borg	14.93 ± 1.44	13.48 ± 1.81	0.01	14.33 ± 1.85	13.63 ± 1.80	0.17
RER rest	0.86 ± 0.06	0.85 ± 0.11	0.74	0.85 ± 0.06	0.86 ± 0.11	0.75
RER AT	0.91 ± 0.08	0.92 ± 0.07	0.95	0.92 ± 0.07	0.92 ± 0.07	0.92
RER peak	0.99 ± 0.09	0.99 ± 0.10	0.95	1.00 ± 0.06	0.99 ± 0.10	0.70
BR peak (%)	53.00 ± 16.19	62.83 ± 12.83	0.02	54.72 ± 15.62	62.79 ± 13.09	0.04
VE AT (L/min)	26.64 ± 6.07	25.30 ± 5.76	0.46	25.65 ± 5.42	25.60 ± 6.02	0.98
VE peak (L/min)	33.13 ± 8.40	32.70 ± 9.59	0.88	30.94 ± 10.18	33.58 ± 8.84	0.31

WR, work rate; HR, heart rate; AT, anaerobic threshold; SBP, systolic blood pressure; DBP, diastolic blood pressure; VO2, oxygen uptake; CO, cardiac output; VE, ventilation; VCO2, carbon dioxide output; OUES, oxygen uptake efficiency slope; DP, heart rate and blood pressure product; RER, respiratory exchange rate.

### The effectivity and safety of CPET

The low-level CPET was aimed at evaluating the exercise capacity and cardiopulmonary function to guide the CR in AMI patients so that it was considered effective as long as AT occurred. In this study, all patients arrived at AT in the low-level CPET, and only one patient had chest pain with ST-segment elevation during the recovery period of CPET.

## Discussion

CPET was an important way to evaluate the cardiopulmonary function and exercise capacity in CR for AMI patients. In this study, AMI patients in stable status performed the low-level CPET at different times. We found that AMI patients had indifferent cardiopulmonary function and exercise capacity regardless of different CPET's time. Patients with complete revascularization and higher LVEF showed better sympathetic regulation and respiratory reserve during exercise. It was safe and effective to conduct the low-level CPET in stable AMI patients 3 days after onset, and complete revascularization and normal LVEF should be good for exercise test in AMI patients.

Senaratne (23) reported the shortest time of CPET was 3 days after AMI that ruled out patients with resting angina pectoris, or uncontrolled HF, or arrhythmia. Goto (20) reported that the exercise test was completed 7 days after AMI in 6 centers and on 14 days after AMI in 7 centers without adverse cardiovascular events. Sivarajan (24) performed the low-level exercise test 1 day before discharge (mean hospital stay 10 days) in AMI. Kunz (25) performed the sub-maximal CPET 22 ± 4 days after AMI. Hamm (26) conducted an exercise test survey indicated that 76% of researchers performed the test within 14 days after AMI, and 24% between 15 and 28 days after AMI. In this study, all patients conducted the CPET from 3 to 15 days after AMI. Apart from VO2/HR at rest, patients in 3–6 days group and 7–9 days group and 10–12 days group and 13–15 days had no statistically different CPET variables. Because of excluding patients with CPET contraindications and AMI complications, all patients were effective to conduct the CPET 3 days after AMI.

Kunz (25) verified that the safety and high sensitivity of the sub-maximal CPET in AMI in evaluating the cardiopulmonary function. Patients with AMI had lower activity tolerance and DP than healthy people (25). Leroy (19) demonstrated that the exercise test could better conduct the risk stratification of patients with AMI that even exceeding the value of coronary angiography. Further, multivariate analysis showed that peak DP and peak HR could be used as predictors of cardiovascular death. Caires (27) also found that ischemia during the exercise test and inappropriate rising in systolic blood pressure were associated with malignant prognosis in AMI. The European Society of Cardiovascular Disease Prevention and Rehabilitation and the American College of Cardiology CPET recommendation pointed out that the VO2 at AT and peakVO2 and VE/VCO2 slope were important indicators for assessing the prognosis of the disease (28). In this study, VO2/HR at rest in 3–6 days group and 10–12 days group were higher than in 13–15 days group, but VO2 at AT, peakVO2, peak DP, peak HR, and VE/VCO2 slope had no statistical difference among the four groups. VO2/HR as surrogates for stroke volume was used to indicate the cardiac output with HR response to exercise to detect cardiac dysfunction (29). Patients in 3–6 days group and 10–12 days group had a better basic cardiac function, but all patients had indifferent cardiopulmonary function and exercise capacity and prognosis during exercise. Furthermore, patients with complete revascularization had higher peak DP and peak BR, and patients with LVEF >50% also had higher peak BR. Complete revascularization was good for coronary blood supply to cardiac contraction and gas exchange during exercise. CPET was a useful method to accurately assess exercise capacity and guide exercise rehabilitation in the clinic.

As early as 1989, Hamm (26) confirmed the safety of exercise test in AMI. The adverse cardiovascular events of exercise test were low in AMI: 0.03% fatal events, and 0.09 % serious non-fatal events, and 1.4% other cardiac complications (26). The aforementioned article mentioned that the exercise test on 3 days after AMI showed 9% positive ST changes, 6.5% minor complications, such as decreased systolic blood pressure, chest pain >5 min without cardiac arrest, 5.5% severe chest pain, and 0.5% non-sustained ventricular tachycardia (23). In this study, AMI patients when were stable performed CPET 3 days after onset without malignant events. Therefore, CPET was safe and feasible 3 days after AMI in stable status.

Goto (20) displayed that 132 AMI patients developed the sub-acute thrombosis within 1 month in 13,685 AMI patients, but only one patient had stent thrombosis related to the exercise test. In this study, only 1 male patient who was diagnosed as acute anterior myocardial infarction had chest pain with ST-segment elevation during the recovery period of the low-level CPET 5 days after AMI. The emergency coronary angiography confirmed the acute stent thrombosis. Nevertheless, the coronary dissection of the left anterior descending vessel had exited in the first emergency coronary angiography. Speculated that the CPET might accelerate the dissection progress, but not the root cause of the stent thrombosis.

The symptom-restricted CPET in AMI had a better predictive value for cardiovascular event risk (30), but had a higher incidence of angina pectoris and ST-segment depression (18). Compared with the symptom-restricted CPET, the low-level CPET might decrease the predicted value but could guarantee the safety that decreased the rate of cardiac complications by 1.9 times (26). In this study, the low-level CPET, namely the RAMP10W scheme, was used to accurately determine the AT and make the individual and scientific guidance for early CR in AMI. More research in the future could explore the safety and predictive value of the symptom-restricted CPET in the acute phase after AMI.

## Limitation

This study was a cross-sectional study, but not a randomized controlled trial. To conform to the inclusion criteria, the sample size of selected patients those from in-patients was relatively small. In this study, CPET was performed on the low-level to decrease the rate of adverse cardiovascular complications, however, the symptom-restricted CPET may be conducted on the premise of comprehensive assessment and guaranteed security in AMI. And CPET variables could be used to assess the prognosis of patients with AMI, but the follow-up of patients with AMI had not been performed in this study. The role of CPET in the prognosis of AMI was to be further studied in future.

## Conclusion

The low-level CPET is safe and efficient to evaluate the exercise capacity in patients with stable AMI patients 3 days after onset. Different times showed no effect on cardiopulmonary function and exercise capacity and prognosis in AMI during CPET. Complete revascularization and normal LVEF should be good for exercise test and exercise training in AMI.

## Data availability statement

The original contributions presented in the study are included in the article/supplementary material, further inquiries can be directed to the corresponding author.

## Ethics statement

The studies involving human participants were reviewed and approved by Beijing Tsinghua Changgung Hospital Research Ethics Committee. The patients/participants provided their written informed consent to participate in this study. Written informed consent was obtained from the individual(s) for the publication of any potentially identifiable images or data included in this article.

## Author contributions

PZ contributed to conception and interpretation. LZ, YL, and SL contributed to conception and design and draft manuscript and acquisition analysis. LZ and SL contributed to perform experiments and interpretation and analyze data. TL and YY critically revised the manuscript and interpretation. FS, RH, YXu, and YXi contributed to perform experiments. All authors reviewed the manuscript.

## References

[1] Haiyan Xu WL Yang J. The China Acute Myocardial Infarction (CAMI) registry: a national long-term registry-research-education integrated platform for exploring acute myocardial infarction in China. Am Heart J. (2016) 175:193–201. 10.1016/j.ahj.2015.04.014 27179740

[2] Rehabilitation AAoCaP. Guidelines for Cardiac Rehabilitation and Secondary Prevention Programs (fifth edition). Human Kinetics (2013).

[3] Group JJW. Guidelines for rehabilitation in patients with cardiovascular disease (JCS 2012). Circulat J. (2014) 78:2022–93. 10.1253/circj.CJ-66-0094 25047729

[4] Thomas RJ Balady G Banka G Beckie TM Chiu J Gokak S. 2018 ACC/AHA clinical performance and quality measures for cardiac rehabilitation: a report of the American college of cardiology/American heart association task force on performance measures. Circ Cardiovasc Qual Outcomes. (2018) 11:e000037. 10.1161/HCQ.0000000000000037 29599285

[5] Piepoli MF Hoes AW Agewall S Albus C Brotons C Catapano AL. 2016 European Guidelines on cardiovascular disease prevention in clinical practice. Eur Heart J. (2016) 37:2315–81. 10.1093/eurheartj/ehw106 27222591 PMC4986030

[6] Ribeiro F Oliveira NL Silva G Campos L Miranda F Teixeira M. Exercise-based cardiac rehabilitation increases daily physical activity of patients following myocardial infarction: subanalysis of two randomised controlled trials. Physiotherapy. (2017) 103:59–65. 10.1016/j.physio.2015.12.002 27012822

[7] Schwaab B Zeymer U Jannowitz C Pittrow D Gitt A. Improvement of low-density lipoprotein cholesterol target achievement rates through cardiac rehabilitation for patients after ST elevation myocardial infarction or non-ST elevation myocardial infarction in Germany: results of the PATIENT CARE registry. Eur J Prev Cardiol. (2019) 26:249–58. 10.1177/2047487318817082 30509144

[8] Dun Y Thomas RJ Smith JR Medina-Inojosa JR Squires RW Bonikowske AR. High-intensity interval training improves metabolic syndrome and body composition in outpatient cardiac rehabilitation patients with myocardial infarction. Cardiovasc Diabetol. (2019) 18:104. 10.1186/s12933-019-0907-0 31412869 PMC6694483

[9] Doll JA Hellkamp A Thomas L Ho PM Kontos MC Whooley MA. Effectiveness of cardiac rehabilitation among older patients after acute myocardial infarction. Am Heart J. (2015) 170:855–64. 10.1016/j.ahj.2015.08.001 26542492

[10] Paolillo S Agostoni P. Prognostic role of cardiopulmonary exercise testing in clinical practice. Ann Am Thorac Soc. (2017) 14:53–8. 10.1513/AnnalsATS.201610-818FR 28362512

[11] Khan H Jaffar N Rauramaa R Kurl S Savonen K Laukkanen JA. Cardiorespiratory fitness and non-fatalcardiovascular events: a population-based follow-up study. Am Heart J. (2017) 184:55–61. 10.1016/j.ahj.2016.10.019 27892887

[12] Lim FY Yap J Gao F Teo LL Lam CSP Yeo KK. Correlation of the New York Heart Association classification and the cardiopulmonary exercise test: a systematic review. Int J Cardiol. (2018) 263:88–93. 10.1016/j.ijcard.2018.04.021 29678511

[13] Vignati C Morosin M Fusini L Pezzuto B Spadafora E De Martino F. Do rebreathing manoeuvres for non-invasive measurement of cardiac output during maximum exercise test alter the main cardiopulmonary parameters? Eur J Prev Cardiol. (2019) 26:1616–22. 10.1177/2047487319845967 31023097

[14] Wasserman K Hansen JE Sue DY Whipp BJ Froelicher VF. 5th principles of exercise testing and interpretation. J Cardiopulmonary Rehabilit Prevent. (2012) 7:189. 10.1097/00008483-198704000-00014

[15] Santiago de. Araújo Pio C, Marzolini S, Pakosh M, Grace SL. Effect of cardiac rehabilitation dose on mortality and morbidity: a systematic review and meta-regression analysis. Mayo Clin Proc. (2017) 92:1644–59. 10.1016/j.mayocp.2017.07.019 29101934

[16] Zhang YM Lu Y Tang Y Yang D Wu HF Bian ZP. The effects of different initiation time of exercise training on left ventricular remodeling and cardiopulmonary rehabilitation in patients with left ventricular dysfunction after myocardial infarction. Disabil Rehabil. (2016) 38:268–76. 10.3109/09638288.2015.1036174 25885667

[17] Rauch B Davos CH Doherty P Saure D Metzendorf M-I Salzwedel A. The prognostic effect of cardiac rehabilitation in the era of acute revascularisation and statin therapy: a systematic review and meta-analysis of randomized and non-randomized studies – The Cardiac Rehabilitation Outcome Study (CROS). Eur J Prev Cardiol. (2016) 23:1914–39. 10.1177/2047487316671181 27777324 PMC5119625

[18] Juneau M Colles P Theroux P de Guise P Pelletier G Lam J. Symptom-limited vs. low level exercise testing before hospital discharge after myocardial infarction. J Am Coll Cardiol. (1992) 20:927–33. 10.1016/0735-1097(92)90195-S 1527304

[19] Leroy F Lablanche JM Bauters C Bertrand ME. Long-term prognosis after myocardial infarction. Value of exercise test compared to clinical features and coronarography. Arch des Maladies du coeur et des Vaisseaux. (1991) 84:485–91. 2064510

[20] Goto Y Sumida H Ueshima K Adachi H Nohara R Itoh H. Safety and implementation of exercise testing and training after coronary stenting in patients with acute myocardial infarction. Circ J. (2002) 66:930–6. 10.1253/circj.66.930 12381088

[21] Casazza F Capozi A Bongarzoni A. Heart rupture during pre-discharge stress test after myocardial infarction. Ital Heart J Suppl. (2001) 2:312–5. 11307790

[22] Kodaira M Itoh T Koizumi K Numasawa Y. Left ventricular free-wall rupture that occurred during a cardiopulmonary exercise test. BMJ Case Rep. (2018) 2018:bcr-2017. 10.1136/bcr-2017-222742 29367222 PMC5786899

[23] Senaratne MP Smith G Gulamhusein SS. Feasibility and safety of early exercise testing using the Bruce protocol after acute myocardial infarction. J Am Coll Cardiol. (2000) 35:1212–20. 10.1016/S0735-1097(00)00545-3 10758963

[24] Sivarajan ES Bruce RA Almes MJ Green B Belanger L Lindskog BD. In-hospital exercise after myocardial infarction does not improve treadmill performance. N Engl J Med. (1981) 305:357–62. 10.1056/NEJM198108133050701 7019706

[25] Kunz VC Serra KB Borges EN Serra PE Silva E. Cardiopulmonary exercise testing in the early-phase of myocardial infarction. Revista brasileira de fisioterapia. (2012) 16:396–405. 10.1590/S1413-35552012005000047 23032293

[26] Hamm LF Crow RS Stull GA Hannan P. Safety and characteristics of exercise testing early after acute myocardial infarction. Am J Cardiol. (1989) 63:1193–7. 10.1016/0002-9149(89)90177-X 2711988

[27] Caires G Mendes M Mesquita A Brizida L Seabra-Gomes R. Value of exercise test for risk stratification acute myocardial infarction. Acta Med Port. (1998) 11:831–8. 10021777

[28] Guazzi M Arena R Halle M Piepoli MF Myers J Lavie CJ. 2016 Focused update: clinical recommendations for cardiopulmonary exercise testing data assessment in specific patient populations. Circulation. (2016) 133:e694–711. 10.1161/CIR.0000000000000406 27143685

[29] Chaudhry S Arena R Bhatt DL Verma S Kumar N. A practical clinical approach to utilize cardiopulmonary exercise testing in the evaluation and management of coronary artery disease: a primer for cardiologists. Curr Opin Cardiol. (2018) 33:168–77. 10.1097/HCO.0000000000000494 29240566 PMC5811236

[30] Jain A Myers GH Sapin PM O'Rourke RA. Comparison of symptom-limited and low level exercise tolerance tests early after myocardial infarction. J Am Coll Cardiol. (1993) 22:1816–20. 10.1016/0735-1097(93)90763-Q 8245334

